# Pulmonary mucormycosis masquerading as endobronchial tumour in an immunocompetent pregnant young lady

**DOI:** 10.1002/rcr2.704

**Published:** 2020-12-23

**Authors:** Khai Lip Ng, Nai‐Chien Huan, Mona Zaria Nasaruddin, Noorul Afidza Muhammad, Ummi Nadira Daut, Jamalul Azizi Abdul Rahaman

**Affiliations:** ^1^ Department of Pulmonology Serdang Hospital Kajang Malaysia; ^2^ Department of Medicine Universiti Putra Malaysia Kajang Malaysia

**Keywords:** Endobronchial tumour, immunocompetent, mucormycosis, pregnant

## Abstract

Pulmonary mucormycosis is a rare but rapidly progressing and life‐threatening fungal infection, usually affecting immunocompromised patients. We report a case of a previously healthy young lady who presented with prolonged cough, weight loss, and haemoptysis. Imaging showed left hilar mass with infiltration into the left main bronchus and concurrent mediastinal lymphadenopathy. Flexible bronchoscopy revealed an endobronchial mass occluding the left main bronchus. Tumour debulking was performed using rigid bronchoscopy with cryoprobe and snares. Histopathological examination revealed inflamed tissue with fungal organism. Fungal polymerase chain reaction (PCR) confirmed *Rhizopus microsporus*. She was treated with two weeks of intravenous amphotericin‐B with complete clinical and radiological resolution.

## Graphical abstract

We report a rare case of pulmonary mucormycosis in an immunocompetent young lady who presented with left hilar mass with endoluminal extension, later found to have *Rhizopus microsporus* infection with multimodality approach. She was successfully treated with antifungal therapy.

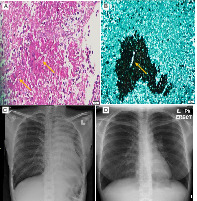

## Introduction

Mucormycosis is a common form of fungal infection among immunocompromised patients [1, 2]. Owing to its angio‐invasive nature, infection can progress rapidly leading to substantial morbidity and mortality [2]. Rhizopus is the most common genus of mucormycosis that is responsible for human infections, accounting for 70–80% of all cases [3]. Seven distinct clinical presentations of mucormycosis have been described, namely: rhino‐orbital‐cerebral, cutaneous, pulmonary, gastrointestinal, isolated central nervous system, renal, and disseminated disease [4]. Patients with haematological malignancies, post‐transplant patients, and patients with poorly controlled diabetes mellitus are particularly at risk of contracting the disease [3, 4, 5]. Rhizopus infection masquerading as endobronchial tumour is a rare clinical entity among immunocompetent patients. Here, we report a young pregnant lady with pulmonary mucormycosis masquerading as left hilar mass with endobronchial extension, leading to considerable diagnostic and therapeutic challenges. She was successfully managed with multimodality approach, incorporating endobronchial interventions and medical therapy.

## Case Report

A 31‐year‐old lady presented with a one‐month history of chronic cough, loss of weight, loss of appetite, and occasional haemoptysis (blood streaks in sputum). She reported no fever, night sweats, skin lesions, eye pain, headache, or symptoms suggestive of rhinitis. She had no known sick contact. There was no recent travelling and she is a lifetime non‐smoker. She is a housewife staying in a terrace house and does not have any pets. Vital signs were stable and she did not require any supplementary oxygen. Physical examination was otherwise unremarkable other than reduced breath sound on the left lung with deviation of trachea to the left.

Her blood and sputum cultures, including Mycobacterium and sputum fungal culture, were negative. C‐reactive protein (CRP) was 14.5 mg/L and erythrocyte sedimentation rate (ESR) was 65 mm/h, while her retroviral screening was negative. White blood cell count was normal at 6.9 × 10^9^/L. Fasting blood sugar was within the normal range. She was found to be seven weeks pregnant during this encounter.

**Figure 1:**
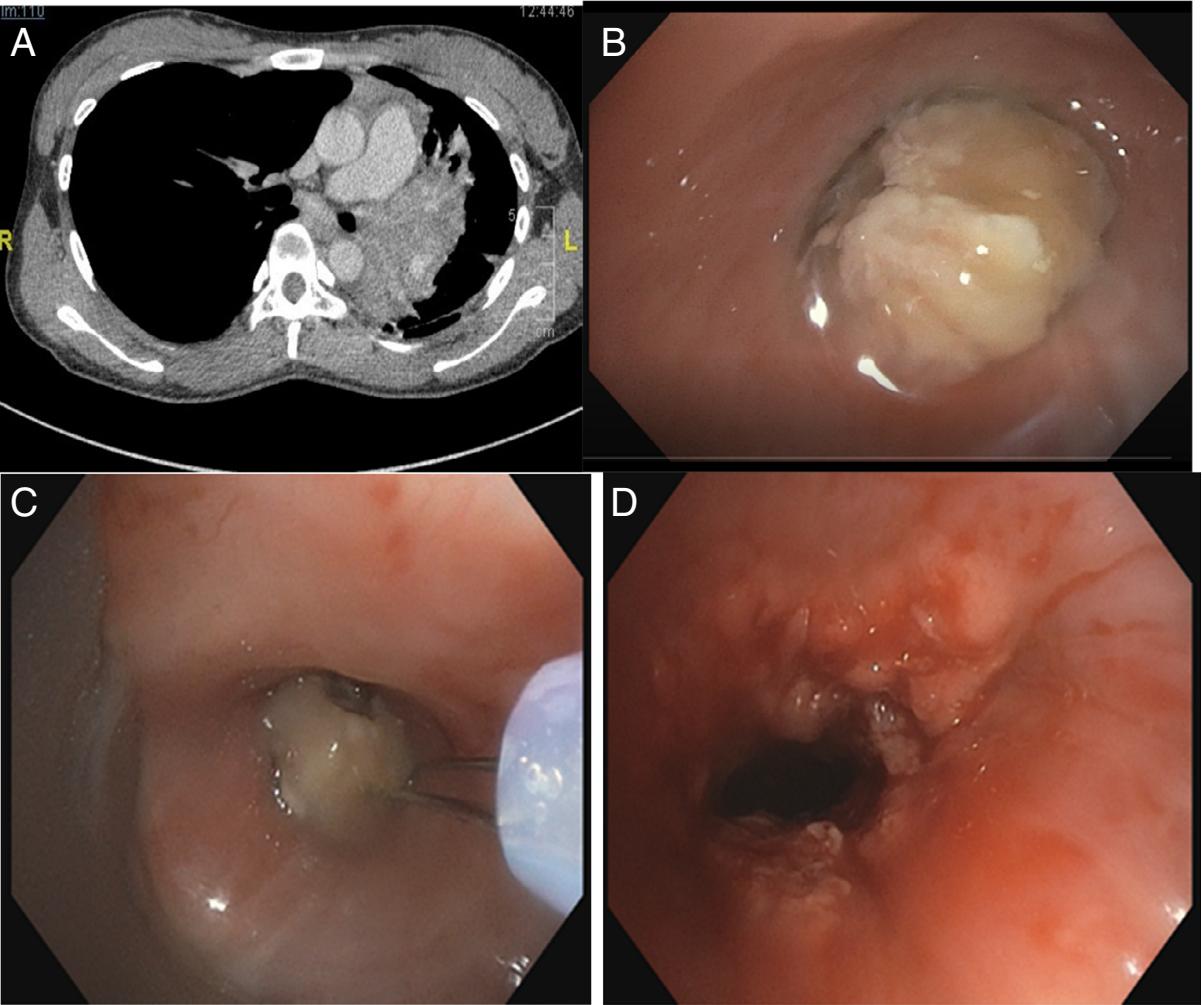
Computed tomography and serial bronchoscopy images. (A) Computed tomography of thorax showed left hilar mass with infiltration into the left main bronchus and mediastinal lymphadenopathy. (B) Endoluminal mass at the left main bronchus. (C) Snaring of endoluminal mass. (D) Post intervention image showing unhealthy and irregular mucosa.

Chest radiograph showed left‐sided homogenous opacity with tracheal deviation to the left side. Bedside ultrasound showed collapsed left lung. Computed tomography showed left hilar mass with infiltration into the left main bronchus and mediastinal lymphadenopathy (Fig. 1A).

Flexible bronchoscopy done revealed a whitish endobronchial mass completely occluding the left main bronchus, about 2 cm from the carina (Fig. 1B). After proper counselling, rigid bronchoscopy was performed under general anaesthesia using bronchial tube, intubated into the left main bronchus. Endobronchial snaring was done (Fig. 1C). After removing the mass obstructing the left main bronchus, copious amount of secretions was observed along distal airways of the left upper and lower lobes. Unhealthy mucosa with multiple nodules was seen along the airway. Cryobiopsy of the nodules was done and argon plasma coagulation was applied on the unhealthy mucosa. Left bronchial airways were patent after procedure (Fig. 1D). The provisional diagnosis at that time was lung malignancy.

**Figure 2:**
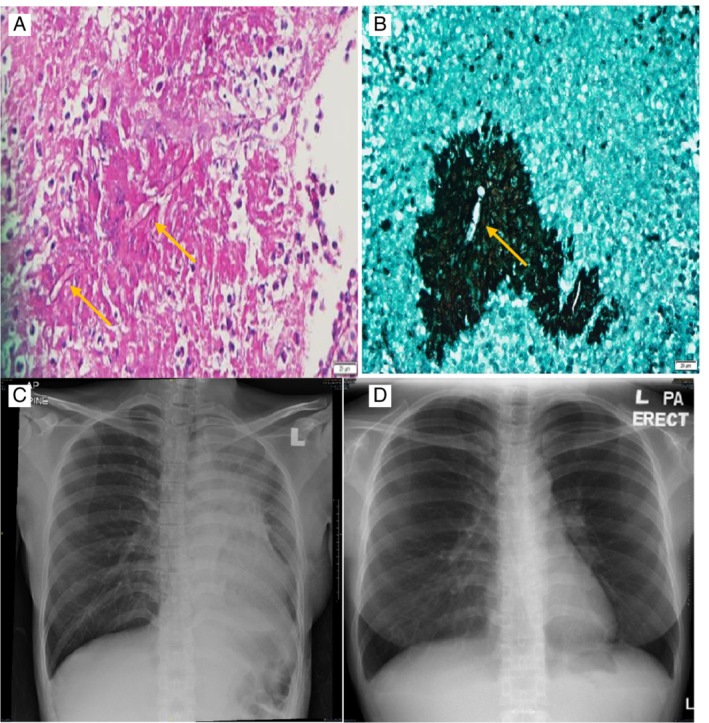
Serial chest radiographs and histopathological images. (A, B) Presence of fungal bodies (yellow arrows) as seen on microscopic images of resected endobronchial mass. (C, D) Serial chest radiograph (pre‐procedure, C, and post completion of intravenous antifungal, D) demonstrating complete resolution of the left lung homogenous opacity.

Endobronchial biopsy later revealed inflamed tissue with the presence of fungal organism. There was no granuloma and malignancy seen. The appearance favours mucormycosis/zygomycosis (Fig. 2A, B). Fungal polymerase chain reaction (PCR) shows *Rhizopus microsporus*. The patient was then treated with intravenous amphotericin‐B for two weeks. Posaconazole was not given as it is classified as class C in pregnancy. Repeated chest radiography after two weeks shows complete resolution of the mass (Fig. 2C, D).

## Discussion

Lymphoma, thymoma, thyroid mass, lung carcinoma, sarcoidosis, and teratoma can all manifest as hilar masses on chest radiographs. In our case report, the aggressive behaviour of left hilar mass together with its invasive properties (i.e. infiltration and obstruction of the left main bronchus) led to a provisional diagnosis of malignancy after initial assessment. Bronchoscopic findings of total occlusion of the left main bronchus by a necrotic mass together with the presence of multiple irregular nodules and mass post debulking further increased our suspicion that malignancy was the most likely diagnosis. To our surprise, fungal bodies were seen under the microscope during histopathological examination of endobronchial tissue samples. Malignant cells were not seen. A confirmatory diagnosis of pulmonary mucormycosis caused by *R*. *microsporus* was made by using fungal PCR.

Mucormycosis is the second most frequent mould infection in immunocompromised patients and can progress rapidly in both immunocompromised and immunocompetent individuals [1, 2]. The angio‐invasive nature of the organism makes early diagnosis and treatment very important [2]. *Rhizopus microsporus* is commonly found in soil, plant debris, and food products such as rice and fermented food [3, 5]. We attempted to identify any possible environmental exposure in our case. Our patient is a housewife. Thorough questioning has failed to reveal any possible association between environmental factors and her infection. Diagnostic evaluation of Rhizopus infection frequently requires an invasive procedure to obtain tissue samples [6]. Sputum and bronchial alveolar lavage failed to reveal any fungal growth in the patient. She had to undergo rigid bronchoscopy with debulking of the intraluminal mass to obtain adequate tissues for identifying fungal body. Advanced laboratory diagnostic testing using fungal PCR was needed to confirm *R*. *microsporus* [3, 6].

Majority of published studies recommend intravenous lipid formulations of amphotericin‐B (5 mg/kg/day) or amphotericin‐B deoxycholate (0.7–1 mg/kg/day) or oral posaconazole 400 mg twice a day for the treatment of pulmonary mucormycosis [7, 8, 9]. Pregnancy status is one of the considerations when selecting antifungal therapy. Intravenous amphothericin‐B (pregnancy risk class B) was selected over oral posaconazole (pregnancy risk class C) in this patient. Amphotericin‐B deoxycholate was used rather than the liposomal form in this case, and the patient did not experience any side effects such as fever, chills, or adverse gastrointestinal reactions. She was treated for two weeks of intravenous antifungal therapy, given via central venous catheter. As there was complete resolution of radiological changes after treatment with intravenous amphotericin‐B for two weeks, the decision was to discontinue the antifungal therapy.

Although rare, mucormycosis has been reported among immunocompetent patients. Mignogna et al. published a case series involving five immunocompetent patients with mucormycosis of maxillary sinuses while Lee et al. reported a case of pulmonary mucormycosis in a patient with chronic obstructive pulmonary disease using inhaled corticosteroid [10, 11]. Ramirez et al., on the other hand, reported a pregnant lady with diabetes mellitus who developed pulmonary mucormycosis in her third trimester of pregnancy [12]. She was subjected to intravenous amphotericin‐B followed by right middle and lower lobectomy due to extensive disease. Our patient, however, was fortunately successfully managed with endoscopic procedures together with intravenous antifungal agent without requiring emergency airway surgery or lung resection. We further explored on whether our patient's pregnancy status increased her susceptibility to infections. A systematic review conducted by Sappenfield et al. concluded that immune alterations in pregnancy may impair pathogen clearance leading to increased severity of disease for several pathogens such as influenza, malaria, and herpes simplex infection [13]. However, it remains unknown on whether pregnancy can increase the risk of pulmonary mucormycosis.

In conclusion, pulmonary mucormycosis should be considered in young patients with no risk factor for malignancy presented with left hilar mass as part of the differential diagnosis. A more proactive approach to obtain a diagnosis is important as it is potentially curable if treated early. Intravenous amphotericin‐B remains the most recommended antifungal for pregnant patients with mucormycosis.

### Disclosure Statement

Appropriate written informed consent was obtained for publication of this case report and accompanying images.

## References

[1] Fernandez JF , Maselli DJ , Simpson T , et al. 2013 Pulmonary mucormycosis: what is the best strategy for therapy? Respir. Care 58(5):e60–e63. 23107233 10.4187/respcare.02106 PMC4066629

[2] Lee FYW , Mossad SB , and Adal KA . 1999 Pulmonary mucormycosis: the last 30 years. Arch. Intern. Med. 159(12):1301–1309. 10386506 10.1001/archinte.159.12.1301

[3] Gomes MZR , Lewis RE , and Kontoyiannis DP . 2011 Mucormycosis caused by unusual mucormycetes, non‐Rhizopus, ‐Mucor, and ‐Lichtheimia species. Clin. Microbiol. Rev. 24(2):411–445. 21482731 10.1128/CMR.00056-10 PMC3122490

[4] Wang XM , Guo LC , Xue SL , et al. 2016 Pulmonary mucormycosis: a case report and review of the literature. Oncol. Lett. 11(5):3049–3053. 27123061 10.3892/ol.2016.4370 PMC4841004

[5] Muqeetadnan M , Rahman A , Amer S , et al. 2012 Pulmonary mucormycosis: an emerging infection. Case Rep. Pulmonol. 2012:1–3. 10.1155/2012/120809 PMC3530759 23304605

[6] Tsyrkunou AV , Ellison RT , Akalin A , et al. 2014 Multifocal *Rhizopus microsporus* lung infection following brush clearing. Med. Mycol. Case Rep. 6:14–17. 25379391 10.1016/j.mmcr.2014.08.001 PMC4216322

[7] Limper AH , Knox KS , Sarosi GA , et al. 2011 An official American Thoracic Society statement: treatment of fungal infections in adult pulmonary and critical care patients. Am. J. Respir. Crit. Care Med. 183:96–128. 21193785 10.1164/rccm.2008-740ST

[8] Spellberg B , Walsh TJ , Kontoyiannis DP , et al. 2009 Recent advance in the management of mucormycosis: from bench to beside. Clin. Infect. Dis. 48:1743–1751. 19435437 10.1086/599105 PMC2809216

[9] Cornely OA , Alastruey‐Izquierdo A , Arenz D , et al. 2019 Global guideline for the diagnosis and management of mucormycosis: an initiative of the European Confederation of Medical Mycology in cooperation with the Mycoses Study Group Education and Research Consortium. Lancet Infect. Dis. 19(12):e405–e421. 31699664 10.1016/S1473-3099(19)30312-3 PMC8559573

[10] Mignogna MD , Fortuna G , Leuci S , et al. 2011 Mucormycosis in immunocompetent patients: a case‐series of patients with maxillary sinus involvement and a critical review of the literature. J. Infect. Dis. 15(8):e533–e540. 10.1016/j.ijid.2011.02.005 21764345

[11] Lee JS , Kim HC , Park SW , et al. 2013 A case of isolated pulmonary mucormycosis in an immunocompetent host. Tuberc. Respir. Dis. 74(6):269–273. 10.4046/trd.2013.74.6.269 PMC3695309 23814599

[12] Ramirez JL , Urisman A , Kukreja J , et al. 2018 Surgical management of pulmonary mucormycosis in third‐trimester pregnancy. Thorac. Cardiovasc. Surg. Rep. 7(1):e27–e29. 29977735 10.1055/s-0038-1660806 PMC6023714

[13] Sappenfield E , Jamieson DJ , and Kourtis AP . 2013 Pregnancy and susceptibility to infectious diseases. Infect. Dis. Obstet. Gynecol. 2013:752852 10.1155/2013/752852. 23935259 PMC3723080

